# Setting a research agenda to advance maternal, newborn, and child health in Ethiopia: An adapted CHNRI prioritization exercise

**DOI:** 10.7189/13.04010

**Published:** 2023-02-10

**Authors:** Michelle L Korte, Habtamu Teklie, Lisanu Taddesse, Bezawit M Hunegnaw, Delayehu Bekele, Getachew Tolera, Meseret Z Tadesse, Grace J Chan

**Affiliations:** 1 Department of Global Health and Population, Harvard T.H. Chan School of Public Health, Boston, Massachusetts, USA; 2 Ethiopia Public Health Institute, Addis Ababa, Ethiopia; 3 HaSET MNCH research program, Addis Ababa, Ethiopia; 4 Department of Pediatrics and Child Health, St. Paul’s Hospital Millennium Medical College, Addis Ababa, Ethiopia; 5 Department of Obstetrics and Gynecology, St. Paul’s Hospital Millennium Medical College, Addis Ababa, Ethiopia; 6 Ethiopian Ministry of Health, Addis Ababa, Ethiopia; 7 Department of Epidemiology, Harvard T.H. Chan School of Public Health, Boston, Massachusetts, USA; 8 Division of Medical Critical Care, Boston Children’s Hospital, Harvard Medical School, Boston, Massachusetts, USA

## Abstract

**Background:**

Critical to the improvement of maternal, newborn, and child health (MNCH) in Ethiopia – where 14 000 mothers die from pregnancy-, childbirth-, or postpartum-related complications each year – is high-quality research and its effective translation into policy and practice. While Ethiopia has rapidly expanded the number of institutions that train and conduct MNCH research, the absence of a shared research agenda inhibits a coordinated approach to inform critical MNCH policy needs. The HaSET Maternal and Child Health Research Program (MCHRP) conducted a mixed methods formative assessment and prioritization exercise to guide investments in future MNCH research in Ethiopia.

**Methods:**

We adapted the Child Health and Nutrition Research Initiative (CHNRI) method, soliciting 56 priority research questions via key informant interviews. Through an online survey, experts scored these on their ability to generate new, actionable evidence that could inform more effective and equitable MNCH programs in Ethiopia. At a workshop in Addis Ababa, experts scored the questions by answerability and ethics, usefulness, disease burden reduction, and impact on equity. Research priority scores were calculated for both the online survey and workshop scoring and averaged to attain a ranked priority list. We validated and contextualized the results by conducting consensus-building discussions with MNCH experts and two community workshops. In total, approximately 100 participants were involved.

**Results:**

Average research priority scores ranged from 58.4 to 83.7 out of 100.0. The top identified research priorities speak to critical needs in the Ethiopian context: to improve population coverage of proven interventions like integrated community case management (ICCM), family integrated newborn care, and kangaroo mother care (KMC); to better understand the determinants of outcomes like home deliveries, immunization drop-out, and antenatal and postpartum care-seeking; and to strengthen health system and workforce capabilities.

**Conclusions:**

This exercise expanded on the CHNRI methodology by comparing prioritization across different audiences, formats, and criteria. Agreement between both scoring rounds and consensus-building discussions was strong, demonstrating the reliability of the CHNRI method. By sharing this research priority list broadly among researchers, practitioners, and donors, we aim to improve coordinated MNCH evidence generation and translation into policy in Ethiopia.

As good maternal, newborn, and child health (MNCH) are fundamental for social and economic development, quality evidence and its effective translation into policy and program action are critical for the improvement of population health and the attainment of sustainable development goals.

Significant gains have been made globally in the reduction of mortality and morbidity of mothers and children. The under-five mortality rate was halved from 2000 to 2019, falling from 76 to 38 deaths per 1000 live births, while the neonatal mortality rate fell from 30 to 17 per 1000 live births [1]. The maternal mortality ratio has also dropped by 38% globally since 2000 [2]. However, significant progress remains to be made. The vast majority of maternal deaths (94%) occur in developing countries. In 2017 alone, about 295 000 mothers died due to pregnancy and childbirth [2], and 5.2 million children died in 2019 [1].

With an estimated population of 101 million people as of 2020, Ethiopia is the second most populous country in Africa and 12^th^ in the world [3]. Child death in Ethiopia decreased by approximately 75% between 1990 and 2019, from 200 to 51 deaths per 1000 live births, but the reduction in neonatal mortality over the same period was far less, at only 53% (from 59 to 28 deaths per 1000 live births) [1]. Maternal mortality also remains high, having declined from 871 to 401 deaths per 100 000 live births from 2000 to 2017 [3]. With around 14 million children under five years old and approximately four million pregnancies annually, this mortality and morbidity contribution is substantial, and MNCH continues to be an utmost health priority in Ethiopia [4].

MNCH research in Ethiopia, including health systems, epidemiologic, and clinical research, has come a long way. Of the 2170 studies published between 1946 and 2018, 70% were published in the last decade alone [5]. Prioritizing research questions is recommended in order to generate policy recommendations for maximum impact on health outcomes, particularly in developing countries like Ethiopia, with limited funding and research capacity and the need to utilize available resources efficiently [6].

In Ethiopia, however, the method through which the available national MNCH research agenda is populated has not been clearly communicated through publication to reliably guide prioritization and coordinate research activities. In 2016, the Ministry of Health (MoH) conducted an MNCH research prioritization exercise [7]. A revision of the priority research lists was conducted in 2019, and the lists are mainly used by the reproductive, maternal, newborn, and child health (RMNCH) and nutrition advisory counsel thematic groups to produce policy recommendations [8]. The results of this exercise have not been widely taken up by relevant stakeholders, who continue to operate in a fragmented environment. Researchers from different national universities have limited knowledge of the priority lists, and funds are not availed by relevant stakeholders to enable researchers to answer those questions.

Various methods are used globally to prioritize research topics. Some common methods include Essential National Health Research, Combined Approach Matrix, James Lind Alliance method, Council on Health Research Development Method, the Delphi process, and the Child Health and Nutrition Research Initiative (CHNRI) method. Each of these methods has their own advantages and disadvantages in identifying participants, scoring criteria and options, transparency, and identifying research ideas. One common limitation of these methods is their reliance on experts and lack of community consultation.

The HaSET (“happiness” in Amharic) Maternal and Child Health Research Program (MCHRP), a collaborative effort across the MoH Maternal and Child Health Nutrition Directorate, Harvard T.H. Chan School of Public Health, St. Paul's Hospital Millennium Medical College (SPHMMC), and the Ethiopian Public Health Institute (EPHI), undertook a formative assessment in order to fill these gaps. The objective of the formative assessment was to identify priorities for MNCH research, as described in this paper, and capacity building, as described elsewhere [9], in order to ultimately improve MNCH evidence generation and its translation into policy and practice in Ethiopia. This paper describes the process undertaken specifically to identify priorities in MNCH future research studies based on gaps in current and past MNCH research and through multi-stakeholder engagement with program leaders, policy makers, program implementers, researchers, funders, experts, and community members.

## METHODS

HaSET’s research priority-setting exercise took place from April 2020 to June 2021, utilizing an adapted CHNRI approach followed by expert- and community-level consensus discussions to identify priorities in MNCH future research studies in Ethiopia.

We followed the below steps per a typical CHNRI approach [6]: 1) process managers defined the context of the priority-setting exercise; 2) a group of technical MNCH experts was invited to submit research ideas; 3) proposed ideas were consolidated into a systematic list of research questions, with duplicates removed and similar options merged; 4) each research question was scored by experts along set criteria; and 5) research priority scores were computed to generate a ranked list of priority research questions.

### Specifying the context

For our study, we specified the context as the prioritization of investments in research for MNCH to reduce mortality and morbidity and to improve birth, early development, and short- and long-term health outcomes among women, newborns, children, and adolescents in Ethiopia (**Box 1**) [6]. Specifically, this study aims to prioritize research questions that can be feasibly implemented within a field site. It complements existing global and national policy targets to improve MNCH outcomes. The target audience includes researchers in the HaSET program as well as HaSET’s partners and other stakeholders in the country.

Box 1 **Context of HaSET’s maternal, newborn, and child health (MNCH) research priority setting exercise in Ethiopia**

1. Population of interest: investments in health research should contribute to disease reduction and improved health among women, newborns, children, and adolescents in Ethiopia.
2. Areas of concern: this health research should contribute to reductions in the mortality and morbidity among the population of interest, namely by improving birth, early development, and short- and long-term health outcomes.
3. Time frame: the first results (i.e. reaching the research endpoints, translating and implementing them, and achieving a detectable disease burden reduction) are expected in the medium-term, within approximately 5-10 years.
4. Stakeholders: the main groups in society whose values and interests should be respected in setting health research investment priorities include the intended beneficiaries of this research at the community level, as well as researchers who have been substantially involved in conducting, translating, and making policy and programmatic decisions based on MNCH research in Ethiopia.
5. Translation and implementation context: HaSET’s investment strategy will be balanced and diversified among the most highly prioritized research options that are also feasible to implement in a field site in the short term. As the final priority list will be widely circulated, other research stakeholders, including funders, may adopt their own investment and implementation strategies.

### Inviting experts to submit ideas via key informant interviews

To solicit research ideas, we utilized key informant interviews (KIIs), asking participants in an open-ended manner to identify the largest gaps and top priorities in MNCH research in Ethiopia. While other CHNRI studies commonly use surveys administered by email to solicit research options, we leveraged qualitative interviews in order to capture additional detail and context around the ideas proposed, including how they relate to gaps or deficiencies in existing research and challenges around translating research to policy; the KIIs also covered related topics such as challenges in the MNCH research environment and capacity-building opportunities, which are described elsewhere [9].

**Figure 1:**
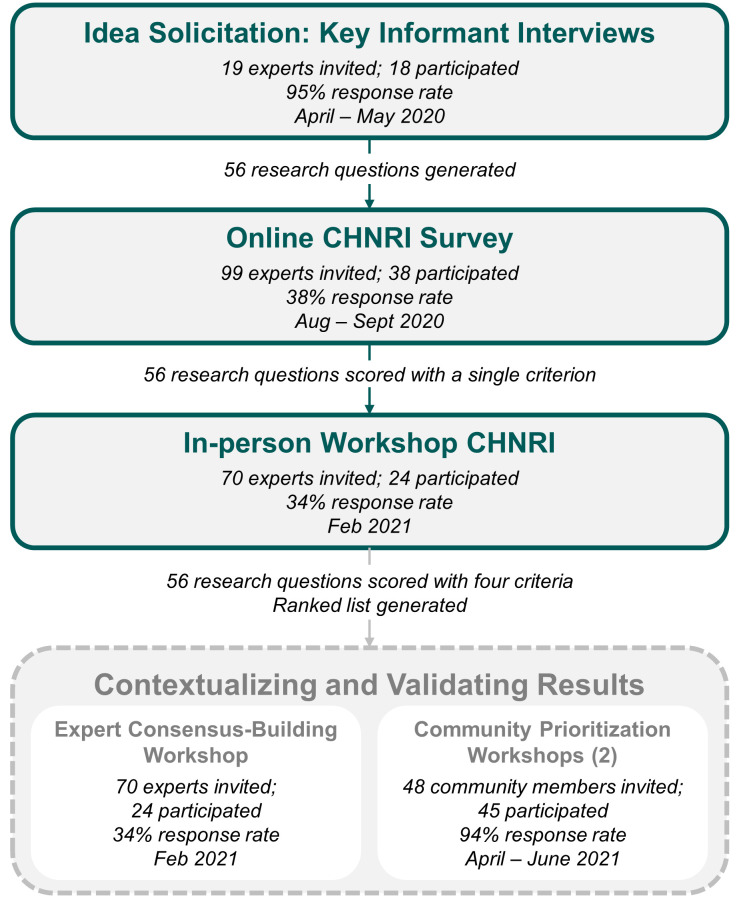
Study design and participation across survey rounds.

Purposive sampling was used throughout the study to select participants based on their expertise and deep knowledge of MNCH activities and research in Ethiopia, including representation from funders of MNCH programs and research, health care workers and association representatives, program implementers from governmental and non-governmental bodies, and academic researchers. We also ensured some participants had expertise from both inside and outside of the country to bring a global perspective to Ethiopia’s MNCH context. **Figure 1** depicts the study design and participation rates across survey rounds.

### Consolidating the research question list

Rapid thematic coding of the KII data was undertaken to identify an initial list of research options. The process managers (MK, LT, BH, HT, GC) reframed these options into research questions, grouping similar responses, filtering for those that satisfied the research context (i.e. research questions that are actionable and answerable within a field site), and adding additional research questions based on their subject matter expertise.

The research questions were categorized into the domains of women’s health, intrapartum period, postpartum period, newborn health, child health, health system, and urban and rural population differences, as these themes were emphasized by KII respondents and help to situate the research questions along the continuum of care.

### Scoring research questions against pre-set criteria

#### Part 1: online survey with single criterion

We posed the resulting list of research questions organized by domain to a wider group of experts, including all who were invited to participate in the KIIs, likewise using purposive sampling to invite participants with diverse expertise in MNCH in Ethiopia. Participants were emailed a description of the study objectives and a link to an electronic survey for scoring.

While CHNRI studies often utilize multiple criteria, to minimize scorer fatigue, we combined multiple criteria into a single composite question for scoring: “Would this research question generate new, actionable evidence that could inform more effective and equitable MNCH programs in Ethiopia?” Participants were asked to use their technical knowledge and expertise to independently score each research question on a sliding scale from 0 to 1, where 0 means “No,” 0.5 means “Undecided,” and 1 means “Yes.” Participants were also given the option to indicate that they are insufficiently informed to score any research question. Lastly, they were given the option to suggest rephrasing any research question.

#### Part 2: in-person workshop scoring with multiple criteria

The process managers refined a few of the research questions based on feedback from the online survey, and the revised list of questions was posed to a group of experts during an in-person workshop. The scoring method was expanded from a single composite criterion to four criteria to gain greater nuance on the strengths and weaknesses of each research question. Participants were invited to score a subset of questions based on their self-identified areas of expertise.

As in prior steps, purposive sampling was used to select participants for their diverse expertise in MNCH research and program implementation in Ethiopia and were invited to attend an in-person workshop in Addis Ababa on February 2-3, 2021. Confirmed attendees were sent pre-reads on research priority setting, and during the workshop, participants attended presentations on MNCH research prioritization experiences and the findings from a scoping review of MNCH studies in Ethiopia [5]. They were introduced to the CHNRI methodology, briefed on the study objectives and methods, and instructed to use a paper scorecard to assign scores for each research question. To score each research question, three sub-questions were presented to assess each of the four criteria. Participants were instructed to independently answer these sub-questions with 0 for “No,” 0.5 for “Undecided,” or 1 for “Yes.” See **Box 2** for the criteria and respective scoring questions, adapted from Rudan (2008) [6].

Box 2 **The four criteria selected for maternal, newborn, and child health (MNCH) research priority-setting and sub-questions questions to assess each criteria**

1. Answerability: the likelihood that the research will indeed reach its proposed endpoints
  1. Would you say the research question is well framed and endpoints are well defined?
  2. Based on: (i) the level of existing research capacity in proposed research; and (ii) the size of the gap from current level of knowledge to the proposed endpoints; would you say that a study can be designed to answer the research question and to reach the proposed endpoints of the research?
  3. Do you think that a study needed to answer the proposed research question would obtain ethical approval without major concerns?
2. Usefulness: the likelihood that the results of the research will generate/improve effective health interventions and results will be delivered to those who need them
  1. Is it likely that the knowledge that would be developed/improved through proposed research would generate or improve effective health interventions?
  2. Taking into account the level of difficulty with implementation from the perspective of the research itself (e.g. design, standardization, safety), the infrastructure required (e.g. human resources, health facilities, communication and transport infrastructure) and users of any applicable intervention (e.g. need for change of attitudes or beliefs, supervision, existing demand), would you say that the endpoints of the research would be deliverable and affordable within the context of interest?
  3. Taking into account government capacity and partnership requirements (e.g. adequacy of government regulation, monitoring and enforcement; governmental intersectoral coordination, partnership with civil society and external donor agencies; favourable political climate to achieve high coverage), would you say that the endpoints of the research would be sustainable within the context of interest?
3. Maximum impact: the likelihood that the research can influence a substantial share of disease cases
  1. Taking into account the results of conducted intervention trials, or for future interventions the proportion of avertable burden under an ideal scenario, would you say that the successful policy and program application of these research findings would have the capacity to remove 5% of disease burden or more?
  2. To remove 10% of disease burden or more?
  3. To remove 15% of disease burden or more?
4. Equity: the likelihood that the results of the research will improve health inequities in the population
  1. Does the present distribution of the disease burden affect mainly the underprivileged in the population?
  2. Would you say that either (i) mainly the underprivileged, or (ii) all segments of the society equally, would be the most likely to benefit from the results of the proposed research after its implementation?
  3. Would you say that the proposed research has the overall potential to improve equity in disease burden distribution in the long term (e.g. 10 years)?

### Calculating research priority scores and generating a ranked list

For the online survey, a research priority score (RPS) was calculated for each research question as the mean score multiplied by 100 to obtain a percentage.

For the in-person scoring using four criteria, intermediate priority scores (IPS) were computed for each criterion for each research question by averaging the three sub-question responses per criterion. Each criterion was given equal weight. The RPS was computed for each research question by averaging these intermediate scores and multiplying by 100 to obtain a percentage [6].

To incorporate the prioritization yielded from both the online survey and in-person CHNRI scoring rounds, for each question we averaged the RPS from each scoring round to generate a single ranked priority list.

### Contextualizing the results

#### Expert consensus-building discussions

Building on the CHNRI scoring exercise, expert discussion groups were convened during the second portion of the in-person workshop. Within each thematic group, a facilitator shared the RPSs from the online survey and preliminary scores from the in-person scoring round, and participants were asked to reflect on these scores and discuss to arrive at a consensus on top research priorities, considering why these are priorities in the Ethiopian MNCH context and how field site researchers could feasibly implement these research questions (e.g. the study design, types and sources of data, resource implications, and specificity of the question). Each group was asked to collectively identify up to five priority research questions per domain, and a notetaker in each group recorded key discussion points, which were shared and discussed in a full plenary. In analysis, the priorities identified via these consensus discussions were compared to the CHNRI priority list.

#### Community prioritization workshops

To further contextualize the CHNRI findings, we solicited input from community members who would be the ultimate beneficiaries of the research questions identified. Two community workshops were held in locations where HaSET regularly conducts field research and maintains established relationships: Angolela and Kewot woredas. Participants were selected via purposive sampling to include women of reproductive age (recruited from the Birhan Maternal and Child Health surveillance cohort) as well as community representatives recruited for the HaSET Community Advisory Board, including local health facility leadership, kebele leaders, religious leaders, and health extension workers.

At each site, participants were divided into four focus groups of 4-8 people each, with one group at each site comprising mothers only to encourage free dialogue. In each group, a facilitator described an MNCH theme (e.g. maternal health, newborn health, child health) and asked participants to brainstorm related issues of greatest importance in their community where new knowledge could produce benefits. Each group brainstormed issues, discussed their rationale, and collectively selected their top 3-5 priorities per theme. Participants then reviewed the top 3 priorities for each theme and collectively agreed on their top 3 overall MNCH priorities across the themes. Throughout these discussions, a notetaker recorded key points to contextualize the priorities identified.

Lastly, the focus groups came together as a plenary to present each group’s top 3 overall MNCH issues, and participants individually ranked their top 5 priorities among these 12 options to arrive at a ranked list of the top MNCH issues by site. For any participants who were illiterate, Birhan staff members assisted them with the ranking by bringing them aside to a private space to discuss their priorities and, as useful, used an exercise of assigning maize kernels to the issues to indicate the participant’s prioritization. The priorities identified in these workshops were reviewed by the process managers, and the top priorities were reframed as research questions and compared to the CHNRI priority list during analysis.

### Data collection

An invitation to inform potential respondents of the study objectives and their requested involvement was emailed or verbally delivered in the case of the community workshops. Informed consent was obtained at the time of data collection, describing precautions to ensure confidentiality, including storing all data in a secure electronic database without personal identifiers throughout data collection, analysis, and reporting the findings.

Semi-structured KIIs were conducted in April-May 2020 by two interviewers with expertise in qualitative research – one native Amharic speaker in Ethiopia who conducted phone interviews and one in-person interview, and one native English speaker in the US (MK) who conducted interviews by phone or Zoom with US-based participants. The interview guide was prepared in English, supposing it would be convenient for the experts (with advanced degrees or above) to describe technical matters. However, if the interviewees preferred to speak in Amharic, the interviewer translated questions to conduct the interview in Amharic. Interviews were conducted at a private and convenient place and time, taking approximately 30 to 60 minutes each. All discussions were tape-recorded and digitally transcribed directly into English via Microsoft Word. At the end of each interview, participant background information was recorded separately.

For the online survey, structured questionnaires were administered electronically via Qualtrics to participants in August-September 2020. Participants indicated consent after presentation with an informed consent script describing the study objectives, methods, and confidentiality measures. Participants were given three weeks to complete the survey, and the research team used follow-up emails and phone calls to encourage participation. Surveys took approximately 30 minutes to complete.

The in-person consensus building workshop took place on February 2-3, 2021, inviting all Ethiopia-based experts who were invited to prior rounds as well as additional MNCH stakeholders. The CHNRI exercise and consensus-building discussions took place on the first day. The second day involved discussions on research capacity-building, as described elsewhere [9].

The community priority-setting workshop in Angolela took place on April 17, 2021, and the workshop in Kewot took place on June 5, 2021. Participants were read a consent script and oriented on the discussion objectives, ground rules, and prioritization procedures, and consent was indicated verbally. Discussions took place in Amharic and were recorded with participants’ consent.

For both the expert and community workshops, considering the potential risk of COVID-19 transmission between data collectors and participants, protocols and training were employed to minimize the risk of transmission and reviewed and approved by EPHI. All data collectors were trained to apply all possible precaution measures, including using masks, keeping physical distance, washing hands with soap and water, and using alcohol-based hand sanitizers. Personal protective equipment was distributed to all participants with instruction to use them at all times. Community workshop discussions were held in outdoor locations, in spaces with open doors and windows to further minimize transmission risks.

All research score calculations were done using Microsoft Excel.

#### Research team

This research was led by the Ministry of Health and HaSET Maternal and Child Research Program in Ethiopia. HaSET was founded in 2018 as an umbrella platform to 1) build local research capacity for MNCH in Ethiopia; 2) identify, prioritize, and conduct MNCH research questions in Ethiopia; and 3) translate evidence to policies and programs to improve maternal and child health. HaSET trains postdoctoral researchers and students and is conducting the first post-doctoral MNCH research fellowship in Ethiopia following a learning-by-doing model where fellows are paired with colleagues at the MoH and lead research studies, conduct secondary data analysis, and translate evidence to policy briefs.

## RESULTS

### Participant demographics

**Figure 2:**
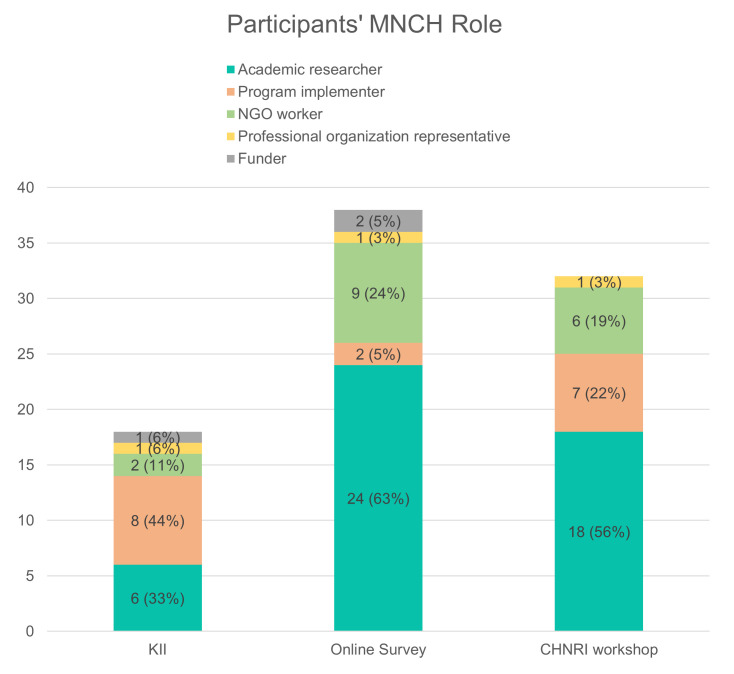
Participant demographics: maternal, newborn, and child health (MNCH) role and expertise.

In each round, participants were asked to self-select into one of the following roles that best describes them: academic researcher, program implementer, non-governmental worker, professional organization representative, or funder (**Figure 2**).

In the input round, 18 of 19 invited MNCH experts participated in KIIs (17 by phone, one in person). Thirteen of the experts identified as male and five as female, and 16 were based in Ethiopia and two outside of Ethiopia.

In the online survey (first scoring round), 38 of 99 invited experts participated in the online survey for a response rate of 38%, which is in line with other CHNRI studies [10]. 66% of online survey respondents identified as male and 34% as female. Thirty were based in Ethiopia and eight outside of Ethiopia.

In the second scoring round, 24 of 70 invited experts participated in the in-person CHNRI scoring for a response rate of 34%, with additional participants joining the subsequent group discussions. 75% of workshop participants identified as male and 25% as female. All participants were based in Ethiopia.

The community prioritization workshops at Angolela and Kewot woredas involved 45 participants: 24 in Angolela and 21 in Kewot. Of these, 10 were mothers recruited from the Birhan MNCH surveillance cohort and 35 were community participants involved in the HaSET Community Advisory Board. Twenty-four identified as male and 21 as female.

### Research priority scores

In total, 56 research questions were generated from analysis and refining of the KIIs, and these were grouped thematically into salient domains across the continuum of care (**Table 1**).

**Table 1:** Research domains and population of interest

Research domain	Population of interest	Total questions n (%)	Top 20 n (%)
Women's health	Women of reproductive age (15-49 years) and pregnant women	12 (21%)	3 (15%)
Intrapartum period	Women and their newborns at childbirth	7 (13%)	3 (15%)
Postpartum period	Postpartum women and their newborns (up to 28 days)	8 (14%)	3 (15%)
Newborn health	Newborns (up to 28 days)	6 (11%)	2 (10%)
Child health	Children in Ethiopia aged 19 years or younger	11 (20%)	6 (30%)
Health system	Women, newborns, children, and adolescents	4 (7%)	3 (15%)
Urban-rural populations	Women, newborns, children, and adolescents	8 (14%)	0 (0%)

The online survey asked participants to score each of the 56 research questions according to the single composite criteria, “Would this research question generate new, actionable evidence that could inform more effective and equitable MNCH programs in Ethiopia?” For the online survey RPS for each of the 56 research questions, see Table S1 in the **Online Supplementary Document**.

The in-person CHNRI scoring round asked participants to score each of the 56 research questions by 12 questions comprising four equally weighted criteria. **Table 2** shows the average IPS for each criteria by research question domain, sorted by the average CHNRI RPS by domain.

**Table 2:** Average intermediate priority scores for child health and nutrition research initiative (CHNRI) criteria by research domain

Research domain	Average CHNRI RPS	Answerability and ethics: average IPS	Usefulness: average IPS	Maximum impact on disease burden: average IPS	Impact on equity: average IPS
Child health	77.26	88.96	84.09	54.51	81.47
Health system	74.57	94.79	80.73	50.37	72.40
Postpartum period	70.56	82.93	72.44	53.36	73.49
Intrapartum period	70.02	79.68	72.88	58.35	69.16
Urban-rural populations	68.56	85.94	76.30	44.05	67.97
Newborn health	67.22	81.25	66.11	46.88	74.65
Women's health	65.36	76.51	64.72	59.70	60.52
Overall	70.51	83.43	73.60	53.33	70.99

CHNRI – child health and nutrition research initiative, RPS – research priority score, IPS – intermediate priority score

To incorporate the prioritization from both the online survey and in-person CHNRI scoring rounds, we averaged the RPS for each scoring round for each research question to generate a single ranked priority list. **Table 3** shows the top 20 research questions ranked by their average RPS, as well as their RPSs from each individual scoring round. For these scores for all 56 research questions, see Table S1 in the **Online Supplementary Document**.

**Table 3:** Average research priority scores, top 20

Domain	Research question	Online survey RPS	CHNRI RPS	Average RPS
Child	Describing the level of effective coverage of ICCM*	83	84.40	83.70
Child	Describing the level of effective coverage of IMNCI*	79	84.70	81.80
Women’s	Identifying qualitative and quantitative factors associated with facility delivery vs home delivery*	79	83.30	81.10
Child	Identifying factors associated with childhood immunization drop-out (including for infants, children, and adolescents)*	80	81.40	80.70
Women’s	Assessing the contribution of maternal diet and nutrition during pregnancy to maternal and newborn outcomes*	81	79.30	80.20
Postpartum	Investigating the effect of family-centered/family-integrated newborn care on postpartum outcomes*	83	77.00	80.00
Postpartum	Identifying barriers to health care-seeking during the postpartum period*	77	82.00	79.50
Health system	Investigating the resiliency of MNCH service delivery/utilization in light of shocks (e.g. pandemics)*	84	74.50	79.20
Women’s	Identifying current behaviors, barriers and supports for ANC adherence*	76	82.20	79.10
Child	Identifying the main factors contributing to morbidity and mortality of children 1 to 59 months of age*	74	83.00	78.50
Postpartum	Describing the level of effective coverage of KMC in facilities and communities*	73	84.00	78.50
Intrapartum	Identifying the main causes of mortality during the intrapartum period*	73	82.90	78.00
Child	Investigating effective strategies to improve girls' and women's empowerment	83	71.40	77.20
Newborn	Investigating the bacterial etiologies of neonatal sepsis and the prevalence of antibiotic resistance*	81	73.20	77.10
Health system	Describing current diagnostic (laboratory and clinical) capacities of the MNCH health workforce*	75	78.10	76.60
Newborn	Identifying the main causes of neonatal mortality and morbidity*	74	78.80	76.40
Intrapartum	Investigating health providers’ ability to correctly identify neonates who need to be resuscitated*	80	72.30	76.10
Intrapartum	Investigating health providers’ adherence to recommendations for vital sign monitoring during labor/delivery	81	70.60	75.80
Health system	Identifying effective strategies for improving health facility documentation of MNCH outcomes such as GA, preterm birth, facility births, hypertension and (pre)eclampsia*	80	71.60	75.80
Child	Investigating factors contributing to immediate initiation and continuation of immunization*	69	81.30	75.10

ICCM – integrated community case management, IMNCI – integrated management of newborn and childhood illness, MNCH – maternal, newborn, and child health, ANC – antenatal care, KMC – kangaroo mother care, GA – gestational age, RPS – research priority score, CHNRI – child health and nutrition research initiative

*Denotes research questions prioritized by the expert consensus discussion groups.

**Table 2** denotes in asterisks the research questions that the expert groups identified as their top priorities via consensus-building discussions. Experts were asked to self-select into one of three groups to score a subset of the 56 research questions (**Table 4**), and some domains were discussed by multiple groups; all of the top priorities identified across groups are depicted. See Table S1 in the **Online Supplementary Document** for the full list of 56 research questions with expert group priorities denoted. Table S2 in the **Online Supplementary Document** displays the top research questions identified by the expert consensus discussions by domain.

**Table 4:** Expert workshop thematic groups

Thematic group	Domains scored and discussed	Members in group
1	Women’s health, intrapartum health, postpartum health	8
2	Intrapartum health, postpartum health, newborn health	8
3	Child health, health systems, urban-rural health differences	8

The community prioritization workshops identified top MNCH issues where additional knowledge would generate the most benefits. See Table S3 in the **Online Supplementary Document** for the full list of the top three issues identified within each of the four themes for each focus group. **Table 5** shows the top five ranked priorities identified per site, reframed as research questions.

**Table 5:** Top maternal, newborn, and child health (MNCH) priorities identified by communities reframed as research questions

Angolela		Kewot	
**Problem / challenge identified**	**Problem framed into research questions**	**Problem / challenge identified**	**Problem framed into research questions**
Limited understanding about the benefits of PNC by the community as well as lack of PNC service provision by health care providers	1. Investigating tools to improve the community and health workers awareness on the benefits of PNC service	Poor quality health service at health facility level	1. Investigating ways to improve the quality of MCH health services at facility level
Several factors are associated with poor early ANC initiation before 16 weeks and follow up	2. Investigating approaches to address the cultural factors that hinder early ANC initiation and ANC follow up	Early marriage and its maternal health-related complications are common in the area	2. Identifying the effects of early marriage and maternal health
Community believes the presence of uvula elongation as a health problem	3. Investigating the incidence of uvulitis and ways to address the malpractice of uvulectomy	Mother’s lack awareness on the benefits of ANC and PNC service	3. Identifying tools to improve mothers’ awareness on the importance of ANC and PNC
Feasible and accurate method of GA estimation for the community	4. Investigating a community-specific, reliable GA measuring tool	Poor ANC follow-up	4. Investigating approaches to improve ANC follow-up
Mothers are not aware of service components provided during facility delivery	5. Investigating approaches to improve mothers’ knowledge on services rendered during facility delivery.	Poor access to health service / facilities	5. Identifying solutions to improve access to MNCH services

PNC – postnatal care, ANC – antenatal care, GA – gestational age, MCH – maternal and child health, MNCH – maternal, newborn, and child health

## DISCUSSION

We selected the CHNRI method given its flexibility in implementation, systematic nature producing an intuitive quantitative score, transparent scoring, and ability to amend stakeholder values over time. With our adaptation of the approach, we were able to compare the prioritization achieved across different rounds and formats and contextualize them with consensus discussions among experts and community members.

Findings from the scoping review of 72 years of MNCH research in Ethiopia show that very few studies included newborns, infants, and postpartum women, even though a significant burden of morbidity and mortality remain in these domains: the most studied populations were children under 10 (30.5%), women of reproductive age (22.0%), and pregnant women (21.9%), while fewer publications looked into newborns (7.0%) and postpartum women (2.9%) [5]. The results of this prioritization exercise follow this trend, with the child health domain most represented among the top 20 research priorities, followed equally by women’s/maternal health, intrapartum period, postpartum period, and health system. None of the urban-rural population questions appeared among the top 20 research priorities.

The highest ranking themes in the domain of child health included studying levels of effective coverage of known interventions and case management packages (ICCM, IMNCI), and reasons for immunization dropout. Similar ranking is reflected in the results of the expert consensus discussions, with additional input that acknowledges the high impact of these packages in newborn and child health care as well as the interest in understanding and improving levels of effective coverage of these high impact intervention packages. While IMNCI has been within the health system for over 20 years and ICCM since 2008, these service delivery packages’ investment return has been in question compared to the success made in reducing newborn and child mortality. This also speaks to the global and national direction toward an effective approach for comprehensive service delivery for community- and facility-level newborn and child health interventions [11].

The prioritized topics in the domain of women’s health included identifying factors associated with location of delivery and antenatal care (ANC) adherence, both of which aim to highlight barriers to facility-based follow-up and care during the critical times of antenatal follow up and delivery. The role of maternal diet and nutrition during pregnancy to pregnancy outcomes was also included in the top 20 priorities. As Ethiopia is a food insecure country, different forms of child, adolescent, and maternal malnutrition may affect pregnancy-related outcomes, in addition to cultural dietary practices. Expert consensus discussions also identified these topics as key priorities, in addition to describing the prevalence of gestational diabetes and investigating the effect of the availability of water, sanitation, and hygiene (WASH) in health facilities on maternal and newborn outcomes.

The top priorities in the postpartum domain included investigating the effect and coverage of low-cost, high-impact interventions such as family-centred newborn care and kangaroo mother care (KMC) as well as identifying barriers to seeking health care during the postpartum period. These topics were prioritized in the expert consensus discussions as well, with emphasis on the need to enable mothers to provide care. Experts additionally identified the description of the psychosocial and mental health characteristics among postpartum women as a research priority in Ethiopia.

Topics prioritized for research in the intrapartum and newborn domains included identification of major causes of morbidity and mortality during these time points. Expert consensus discussions expanded on this to stress the need for more robust study designs to understand the main causes of morbidity and mortality in the different periods in the Ethiopian context to generate accurate data for policy and programmatic activities. With respect to the newborn domain, research to investigate the aetiology and antimicrobial resistance patterns of neonatal sepsis ranked highest both in RPS as well as in the expert consensus discussions, highlighting the gaps in knowledge in this area. Top intrapartum priorities also included investigation of health provider capabilities, including the ability to identify neonates in need of resuscitation and adherence to recommendations for vital sign monitoring during labour, highlighting broader health system needs.

A few research questions surfaced in the top 20 research questions specific to the health system domain, including studies to investigate the resiliency of MNCH service delivery and utilization in light of shocks such as pandemics. This likely arose from current experiences with COVID-19 and the impact on health systems and essential services: essential MNCH services such as family initiation and sick visits for children significantly declined during the COVID-19 pandemic in the Amhara Region [12], and 28% of health care providers surveyed in Addis Ababa reported interruptions in child health services while more than 50% reported interruptions in ANC, folate supplementation, and family planning [13]. Also ranking highly in the health system domain is the description of current laboratory and clinical diagnostic capacities of the MNCH health workforce, which may serve to identify gaps and areas for investment. Additionally, the prioritization exercises also identified poor documentation of MNCH outcomes in health facilities as a major health systems problem and included the need to identify effective strategies to improve health facility documentation of key MNCH outcomes as an important research priority in this domain. This is also expected to improve accurate description and understanding of the burden of key pregnancy, maternal, and neonatal outcomes. Notably, priorities identified in the online survey, CHNRI, and expert consensus discussions all aligned to identify these research questions as the highest priorities within the health system domain.

Overall, the priorities generated in this exercise speak to a few critical needs specific to the Ethiopian context: the need to improve population coverage of proven interventions like ICCM, family integrated newborn care, and KMC; the need to better understand the determinants of outcomes like home deliveries, immunization drop-out, and antenatal and postpartum care-seeking; and the need to strengthen health system and workforce capabilities. They also align with the maternal and newborn health research priorities identified in a continent-wide CHNRI exercise of over 900 experts: the top 10 priorities for East Africa identified in that study included practices to reduce neonatal sepsis, coverage and uptake of KMC, and uptake of essential newborn care [14].

Intermediate priority scores show that the research questions generated and prioritized in this exercise rank most highly in answerability and ethics (83% average IPS) and least highly in achieving the maximum impact on disease burden (53% average IPS). A large portion of the research questions lend themselves to descriptive epidemiological studies, but a number could be pursued as intervention studies with large potential for impact such as investigating the effect of family-centred/family-integrated newborn care on postpartum outcomes; investigating effective strategies to improve girls' and women's empowerment; identifying effective strategies for improving health facility documentation of MNCH outcomes such as gestational age, preterm birth, facility births, hypertension and (pre)eclampsia.

The prioritization by community members supports the expert CHNRI ranking in certain areas. For example, communities identified poor awareness of postnatal care services (and also ANC and delivery services) as key issues, and the CHNRI likewise prioritized understanding the barriers to health care seeking during the postpartum period. Communities raised the importance of studying factors that prevent early initiation of ANC and follow-up, while the CHNRI and online survey results prioritized identifying factors related to adherence to ANC. Additionally, communities highlighted concerns about poor quality of health services and the need for research to understand related gaps. Areas highlighted by communities that did not rank highly or at all in the CHNRI priority list include improving the accuracy of gestational age estimation during pregnancy, concerns about certain community beliefs (such as elongation of the uvula), and understanding the effects and health impacts of harmful traditional practices such as uvulectomy (cutting of the uvula) and early child marriage that are prevalent in the area. These topics were either not reflected or highly prioritized in the 56 research questions, underscoring the importance of community participation in setting agendas and priorities for research that will take place in those communities and is responsive to local health concerns.

### Limitations

This study had two main limitations – the complexity of arriving at a single unambiguous ranking given the differing samples, formats, and criteria used in each round, and the constraining of the context to research questions that could feasibly be implemented within a field site.

While the CHNRI is a flexible method yielding priorities that could be adapted over time via the incorporation of stakeholder input, arriving at a single ranked priority list is useful for the credibility of the exercise and ability to disseminate results. In typical CHNRI studies, a single scoring round is used to generate such a list, while in our study, we used two distinct scoring rounds with different samples, formats, and criteria generating distinct RPSs. As the in-person CHNRI scoring used four criteria, the assigned priority scores may be more targeted and, therefore, more reliable than those from the online survey, which used a broader single criterion. At the same time, the sample for the in-person scoring round was smaller than that of the online survey and more restricted given travel restrictions related to COVID-19, raising the possibility that key voices may have been omitted. To balance the input from both rounds, we calculated the final priority list using a simple average of the RPSs generated from each round. These modifications are supported by the flexibility of the CHNRI method: by 2016, over two-thirds of CHNRI studies had modified the number and focus of criteria [10]. Further, a study using partial criteria scoring found high average expert agreement, suggesting that using fewer criteria may be both a valid and pragmatic option in priority setting exercises [15].

Additionally, limiting the context to research questions that can be feasibly implemented in a field site reduced the scope of research questions and the potential utility of this list for other stakeholders. Namely, the KIIs identified a number of issues around quality of care, and we excluded these given the inability to implement them. The primary study designs that could be implemented by a field site are epidemiological and descriptive studies, and the study designs represented in the research priority list are constrained accordingly. This may affect the extent to which the priority list may be accepted as a national priority list addressing concerns of the MOH and other policymakers, as well as global priorities (e.g. local implementation of global interventions, studying cost-effectiveness).

**Figure 3:**
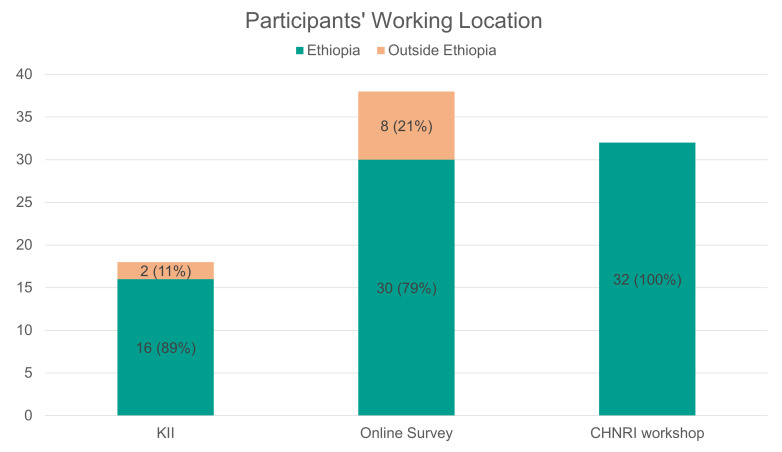
Participant demographics: working location.

**Figure 4:**
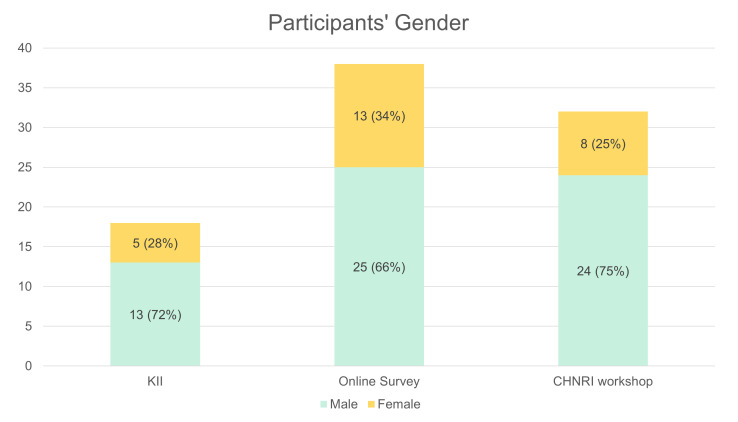
Participant demographics: gender.

We also faced sampling challenges. The online survey sample heavily represented academics, while implementers and health care professional association members comprised a smaller proportion of respondents, limiting the representativeness of frontline perspectives on research needs (**Figure 2**). The expert workshop in Addis Ababa had limited representation of invited experts from regional health bureaus and universities outside of Addis Ababa, likely due to COVID-related travel difficulties (**Figure 3**). Overall, take-up for both online survey and in-person workshops were low but similar to take-up rates of other CHNRI surveys [10]. The gender balance across all data collection rounds was disproportionately skewed toward males (**Figure 4**), reflecting the need to ensure greater gender representation in future efforts.

### Strengths

This study represents one of the first applications of the CHNRI methodology to guide investments in MNCH research in Ethiopia in a transparent, democratic, inclusive, and replicable manner. By engaging a wide variety of MNCH stakeholders, greater ownership and uptake of the results is expected.

Leveraging the CHNRI method’s flexibility, our study design enabled the comparison of RPSs obtained from the different scoring rounds and methods, including when a single composite criterion is used vs four explicit criteria. We also were able to validate the CHNRI approach by leveraging the strengths of discussion-based priority-setting methods and by incorporating community priorities, which are largely under-solicited in research priority-setting techniques. While discussion-based consensus building techniques have limitations, namely the potential bias introduced when scores are not independent and may be influenced by strong opinions in the group, they also have strengths lacking in the CHNRI exercise: the group is able to discuss amongst themselves to clarify potential misunderstandings and refine their interpretations, particularly against a greater collective pool of information and knowledge about the research context, and political will may be built over the course of these discussions that can be beneficial to activate in carrying the final recommendations forward. By utilizing the CHNRI method for the ranked list and indicating where expert consensus was strongest, we are able to provide more information to users of the list and inspect how the expert discussion-based consensus compares to that yielded through the systematic CHNRI process. In this case, the expert consensus tracked strongly to the CHNRI results.

Similarly, the community prioritization workshops confirmed the importance and relevance of the priorities surfaced and ranked by MNCH experts, while highlighting additional context-specific priorities that researchers working in those areas should consider. Future studies may build upon and refine these community priority-setting methods.

### Application of results

The CHNRI method aims to assist policymakers as well as donors in understanding how different research domains and questions may contribute to improved health outcomes. However, a research priority list is only as good as its implementation. Leveraging HaSET’s existing and expanding partnerships in Ethiopia, we anticipate that the results of this adapted CHNRI exercise may influence not only MNCH research over the coming years, but also policy and program decisions with the potential to reduce the MNCH-related disease burden over time.

In the immediate term, research questions identified and prioritized through this exercise will inform the HaSET research fellowship program, which aims to build a generation of MNCH researchers who are supported to conduct independent research as principal investigators. HaSET will also continue to strengthen its partnership with the MoH to support it and other partners in answering specific research questions. HaSET’s research platform, the BIRHAN field site, encompasses approximately 80 000 people living in 20 000 households in 16 villages in rural Ethiopia and includes three hospitals and five health centres. As HaSET collects routine health and demographic data and longitudinal maternal and child health data on exposures and health outcomes throughout the site, it is well-positioned to answer some of the prioritized research questions and continue to provide MNCH data and evidence for the MOH and policy makers in Ethiopia for additional analyses. Additional information about HaSET can be found at www.haset.org. 

These lists should be shared widely with all relevant stakeholders to ensure research take-up, research translation, and ultimately disease burden reduction. Ideally, a variety of actors within the Ethiopian MNCH research ecosystem may work in complement to pursue the prioritized research questions to avoid redundancies, and donors may utilize these lists to prioritize coordinated research funding. Given the flexibility of the CHNRI method, future exercises may be conducted to assign value-based weights to the four criteria and incorporate wider stakeholder input.

## Additional material


Online Supplementary Document
