# Supplementary material for: Setting a research agenda to advance maternal, newborn, and child health in Ethiopia: An adapted CHNRI prioritization exercise
Source: J Glob Health. 2023 Feb 10;13:04010. doi: 10.7189/13.04010 (PMC9910124; doi:10.7189/13.04010)
Supplement: Online Supplementary Document [file jogh-13-04010-s001.pdf]

ONLINE SUPPLEMENTARY DOCUMENT

Title: Setting a Research Agenda to Advance Maternal, Newborn, and Child Health in Ethiopia: an Adapted CHNRI Prioritization Exercise

Authors: Michelle L. Korte, Habtamu Teklie, Lisanu Taddesse, Bezawit M. Hunegnaw, Getachew Tolera, Meseret Z. Tadesse, Grace J. Chan

S1a. Research Priority Scores and Intermediate Priority Scores for all 56 Research Questions, Ranked by Average RPS

No.	*Domain*	*Research question*	*Online Survey RPS*	*1. Answerability and Ethics IPS*	*2. Usefulness IPS*	*3. Disease Burden Reduction IPS*	*4. Impact on Equity IPS*	*CHNRI RPS*	*Average RPS*
35	child	Describing the level of effective coverage of ICCM*	**83.0**	93.8	85.4	69.0	89.6	**84.4**	**83.7**
36	child	Describing the level of effective coverage of IMNCI*	**79.0**	93.8	95.8	59.5	89.6	**84.7**	**81.8**
8	women's	Identifying qualitative and quantitative factors associated with facility delivery versus home delivery*	**79.0**	90.8	84.0	79.9	78.5	**83.3**	**81.1**
38	child	Identifying factors associated with childhood immunization drop-out (including for infants, children, and adolescents)*	**80.0**	89.6	87.5	54.8	93.8	**81.4**	**80.7**
1	women's	Assessing the contribution of maternal diet and nutrition during pregnancy to maternal and newborn outcomes*	**81.0**	92.9	81.8	70.2	72.3	**79.3**	**80.2**
24	postpartum	Investigating the effect of family-centered/family-integrated newborn care on postpartum outcomes*	**83.0**	86.8	80.3	57.1	83.7	**77.0**	**80.0**
22	postpartum	Identifying barriers to health care-seeking during the postpartum period*	**77.0**	93.0	81.2	65.7	88.2	**82.0**	**79.5**
48	health system	Investigating the resiliency of MNCH service delivery/utilization in light of shocks (e.g. pandemics)*	**84.0**	93.8	77.1	50.0	77.1	**74.5**	**79.2**
7	women's	Identifying current behaviors, barriers and supports for antenatal care (ANC) adherence*	**76.0**	93.2	83.6	79.5	72.6	**82.2**	**79.1**
40	child	Identifying the main factors contributing to morbidity and mortality of children 1 to 59 months of age*	**74.0**	91.7	83.3	73.8	83.3	**83.0**	**78.5**
20	postpartum	Describing the level of effective coverage of KMC in facilities and communities*	**73.0**	92.9	91.0	70.6	81.5	**84.0**	**78.5**
13	intrapartum	Identifying the main causes of mortality during the intrapartum period*	**73.0**	82.4	82.5	75.7	91.0	**82.9**	**78.0**
42	child	Investigating effective strategies to improve girls' and women's empowerment	**83.0**	79.2	75.0	50.0	81.3	**71.4**	**77.2**
31	newborn	Investigating the bacterial etiologies of neonatal sepsis and the prevalence of antibiotic resistance*	**81.0**	81.7	77.9	64.6	68.8	**73.2**	**77.1**

45	health system	Describing current diagnostic (laboratory and clinical) capacities of the MNCH health workforce*	**75.0**	97.9	81.3	56.3	77.1	**78.1**	**76.6**
30	newborn	Identifying the main causes of neonatal mortality and morbidity*	**74.0**	85.8	77.1	75.0	77.1	**78.8**	**76.4**
14	intrapartum	Investigating health providers' ability to correctly identify neonates who need to be resuscitated*	**80.0**	84.0	75.8	62.6	66.8	**72.3**	**76.1**
15	intrapartum	Investigating health providers' adherence to recommendations for vital sign monitoring during labor/delivery	**81.0**	85.3	71.8	54.0	71.4	**70.6**	**75.8**
47	health system	Identifying effective strategies for improving health facility documentation of MNCH outcomes such as gestational age, preterm birth, facility births, hypertension and (pre)eclampsia*	**80.0**	95.8	83.3	40.5	66.7	**71.6**	**75.8**
43	child	Investigating factors contributing to immediate initiation and continuation of immunization*	**69.0**	89.6	93.8	56.3	85.4	**81.3**	**75.1**
3	women's	Assessing the contribution of preconception diet and nutrition to maternal and newborn outcomes	**77.0**	95.1	65.3	68.0	57.5	**71.5**	**74.2**
49	urban rural	Describing care-seeking behavior across different geographies (urban and rural)	**68.0**	91.7	91.7	59.5	77.1	**80.0**	**74.0**
12	women's	Investigating the effect of the availability of WASH in health facilities on maternal and newborn outcomes*	**77.0**	86.3	67.9	60.4	68.5	**70.8**	**73.9**
39	child	Identifying the main factors contributing to morbidity and mortality of adolescents (10 to 19 years of age)	**68.0**	89.6	81.3	73.8	64.6	**77.3**	**72.7**
53	urban rural	Describing the usage of over-the-counter, private, and traditional medicines*	**74.0**	75.0	75.0	54.8	77.1	**70.5**	**72.2**
18	intrapartum	Investigating the effect of neonatal resuscitation on child survival and cognitive development	**74.0**	79.8	70.1	58.2	68.3	**69.1**	**71.5**
41	child	Identifying the main factors contributing to morbidity and mortality of children 5 to less than 10 years of age	**67.0**	95.8	77.1	52.1	68.8	**73.4**	**70.2**
5	women's	Describing the prevalence of gestational diabetes*	**66.0**	88.7	77.4	64.6	66.4	**74.3**	**70.1**
34	child	Describing current child immunization coverage levels (including for infants, children, and adolescents)*	**62.0**	91.7	89.6	35.7	91.7	**77.2**	**69.6**
46	health system	Describing the level of health facility readiness, including equipment/material availability for MNCH services*	**65.0**	91.7	81.3	54.8	68.8	**74.1**	**69.6**

51	urban rural	Describing food taboos, including fasting, for pregnant and lactating women across different geographies (urban and rural)*	**71.0**	85.4	70.8	40.5	75.0	**67.9**	**69.5**
52	urban rural	Describing the private health system and urban/rural population differences in seeking care from private health care providers	**71.0**	87.5	72.9	26.2	83.3	**67.5**	**69.2**
6	women's	Developing algorithms to improve the identification of high risk pregnant women	**83.0**	61.9	57.1	47.6	52.4	**54.8**	**68.9**
4	women's	Describing the prevalence and etiologies of conditions such as maternal hypertension, pre-eclampsia, eclampsia, and HELLP	**75.0**	70.2	59.2	56.3	64.3	**62.5**	**68.8**
44	child	Investigating the effect of early child stimulation on development/cognition in the first 2 years of life*	**74.0**	84.8	75.0	24.6	69.0	**63.4**	**68.7**
26	postpartum	Investigating the impact of cultural practices such as postpartum seclusion of mother and infant on maternal and newborn outcomes	**71.0**	78.9	67.0	46.7	72.9	**66.4**	**68.7**
16	intrapartum	Investigating the benefits of monitoring vital signs during the intrapartum period and at what frequency	**74.0**	79.8	63.9	45.1	60.3	**62.3**	**68.1**
28	newborn	Identifying factors and characteristics associated with neonatal growth (weight and length)	**73.0**	77.1	62.9	37.5	68.8	**61.6**	**67.3**
19	intrapartum	Investigating the effectiveness of intrapartum antibiotic prophylaxis in preventing early-onset group B streptococcal disease*	**67.0**	69.6	76.2	56.9	66.3	**67.3**	**67.1**
23	postpartum	Investigating how birth and maternal outcomes compare between home births and facility births	**57.0**	92.7	70.8	66.7	77.7	**77.0**	**67.0**
27	postpartum	Investigating the impact of mother-infant separation in facilities after birth and at subsequent service delivery points in the postpartum period on newborn outcomes	**76.0**	70.5	64.6	41.7	54.9	**57.9**	**67.0**
56	urban rural	Investigating the relationship between air pollution exposure and MNCH outcomes*	**70.0**	85.4	68.8	35.7	64.6	**63.6**	**66.8**
21	postpartum	Describing the psychosocial and mental health characteristics among postpartum women*	**70.0**	83.2	68.6	38.4	63.3	**63.4**	**66.7**
54	urban rural	Investigating formula feeding practices and their effects on newborn outcomes across urban/rural populations*	**63.0**	81.3	77.1	54.8	64.6	**69.4**	**66.2**
29	newborn	Identifying factors associated with immediate and exclusive breastfeeding among women who gave birth at home compared to women who gave birth at a facility	**64.0**	83.8	61.7	33.3	87.5	**66.6**	**65.3**

2	women's	Assessing the contribution of maternal literacy to maternal and newborn outcomes	**57.0**	90.8	69.9	65.5	66.7	**73.2**	**65.1**
17	intrapartum	Investigating the effect of antenatal corticosteroids on neonatal outcomes	**61.0**	76.9	69.8	56.0	60.1	**65.7**	**63.4**
37	child	Describing the prevalence of HIV among children (5 to less than 10 years of age)	**54.0**	79.2	81.3	50.0	79.2	**72.4**	**63.2**
32	newborn	Investigating the effect of maternal survival on neonatal survival	**63.0**	74.2	55.0	33.3	79.2	**60.4**	**61.7**
50	urban rural	Describing current consumption levels of processed foods (e.g. snack foods, soft drinks)	**61.0**	91.7	75.0	26.2	47.9	**60.2**	**60.6**
55	urban rural	Investigating how Cesarean section rates compare between urban and rural populations	**51.0**	89.6	79.2	54.8	54.2	**69.4**	**60.2**
33	newborn	Investigating the effects of immediate and exclusive breastfeeding on newborn outcomes	**56.0**	85.0	62.1	37.5	66.7	**62.8**	**59.4**
9	women's	Identifying the primary social determinants, etiologies, and mechanisms leading to maternal mortality globally, in Ethiopia, or in specific regions of Ethiopia	**76.0**	50.0	38.2	38.9	41.0	**42.0**	**59.0**
11	women's	Investigating the effect of environmental exposures (e.g. organophosphates) during pregnancy on pregnancy, birth, and child outcomes over time	**67.0**	53.9	54.0	46.0	47.3	**50.3**	**58.6**
10	women's	Identifying the significant maternal health conditions and causes of maternal mortality globally, in Ethiopia, or in specific regions of Ethiopia	**77.0**	44.4	38.2	39.6	38.9	**40.3**	**58.6**
25	postpartum	Investigating the efficacy of tranexamic acid for postpartum hemorrhage prevention	**60.0**	65.5	55.9	40.1	65.7	**56.8**	**58.4**

*Note: No. indicates the order in which the research question was presented to respondents. * denote research questions prioritized by the expert consensus discussion groups.*

S1b. Research Priority Scores and Intermediate Priority Scores for all 56 Research Questions, Ranked by Average RPS Within Domain

No.	*Domain*	*Research question*	*Online Survey RPS*	*1. Answerability and Ethics IPS*	*2. Usefulness IPS*	*3. Disease Burden Reduction IPS*	*4. Impact on Equity IPS*	*CHNRI RPS*	*Average RPS*
8	women's	Identifying qualitative and quantitative factors associated with facility delivery versus home delivery*	**79.0**	90.8	84.0	79.9	78.5	**83.3**	**81.1**
1	women's	Assessing the contribution of maternal diet and nutrition during pregnancy to maternal and newborn outcomes*	**81.0**	92.9	81.8	70.2	72.3	**79.3**	**80.2**
7	women's	Identifying current behaviors, barriers and supports for antenatal care (ANC) adherence*	**76.0**	93.2	83.6	79.5	72.6	**82.2**	**79.1**
3	women's	Assessing the contribution of preconception diet and nutrition to maternal and newborn outcomes	**77.0**	95.1	65.3	68.0	57.5	**71.5**	**74.2**
12	women's	Investigating the effect of the availability of WASH in health facilities on maternal and newborn outcomes*	**77.0**	86.3	67.9	60.4	68.5	**70.8**	**73.9**
5	women's	Describing the prevalence of gestational diabetes*	**66.0**	88.7	77.4	64.6	66.4	**74.3**	**70.1**
6	women's	Developing algorithms to improve the identification of high risk pregnant women	**83.0**	61.9	57.1	47.6	52.4	**54.8**	**68.9**
4	women's	Describing the prevalence and etiologies of conditions such as maternal hypertension, pre-eclampsia, eclampsia, and HELLP	**75.0**	70.2	59.2	56.3	64.3	**62.5**	**68.8**
2	women's	Assessing the contribution of maternal literacy to maternal and newborn outcomes	**57.0**	90.8	69.9	65.5	66.7	**73.2**	**65.1**
9	women's	Identifying the primary social determinants, etiologies, and mechanisms leading to maternal mortality globally, in Ethiopia, or in specific regions of Ethiopia	**76.0**	50.0	38.2	38.9	41.0	**42.0**	**59.0**

11	women's	Investigating the effect of environmental exposures (e.g. organophosphates) during pregnancy on pregnancy, birth, and child outcomes over time	**67.0**	53.9	54.0	46.0	47.3	**50.3**	**58.6**
10	women's	Identifying the significant maternal health conditions and causes of maternal mortality globally, in Ethiopia, or in specific regions of Ethiopia	**77.0**	44.4	38.2	39.6	38.9	**40.3**	**58.6**
13	intrapartum	Identifying the main causes of mortality during the intrapartum period*	**73.0**	82.4	82.5	75.7	91.0	**82.9**	**78.0**
14	intrapartum	Investigating health providers' ability to correctly identify neonates who need to be resuscitated*	**80.0**	84.0	75.8	62.6	66.8	**72.3**	**76.1**
15	intrapartum	Investigating health providers' adherence to recommendations for vital sign monitoring during labor/delivery	**81.0**	85.3	71.8	54.0	71.4	**70.6**	**75.8**
18	intrapartum	Investigating the effect of neonatal resuscitation on child survival and cognitive development	**74.0**	79.8	70.1	58.2	68.3	**69.1**	**71.5**
16	intrapartum	Investigating the benefits of monitoring vital signs during the intrapartum period and at what frequency	**74.0**	79.8	63.9	45.1	60.3	**62.3**	**68.1**
19	intrapartum	Investigating the effectiveness of intrapartum antibiotic prophylaxis in preventing early-onset group B streptococcal disease*	**67.0**	69.6	76.2	56.9	66.3	**67.3**	**67.1**
17	intrapartum	Investigating the effect of antenatal corticosteroids on neonatal outcomes	**61.0**	76.9	69.8	56.0	60.1	**65.7**	**63.4**
24	postpartum	Investigating the effect of family-centered/family-integrated newborn care on postpartum outcomes*	**83.0**	86.8	80.3	57.1	83.7	**77.0**	**80.0**
22	postpartum	Identifying barriers to health care-seeking during the postpartum period*	**77.0**	93.0	81.2	65.7	88.2	**82.0**	**79.5**
20	postpartum	Describing the level of effective coverage of KMC in facilities and communities*	**73.0**	92.9	91.0	70.6	81.5	**84.0**	**78.5**

26	postpartum	Investigating the impact of cultural practices such as postpartum seclusion of mother and infant on maternal and newborn outcomes	**71.0**	78.9	67.0	46.7	72.9	**66.4**	**68.7**
23	postpartum	Investigating how birth and maternal outcomes compare between home births and facility births	**57.0**	92.7	70.8	66.7	77.7	**77.0**	**67.0**
27	postpartum	Investigating the impact of mother-infant separation in facilities after birth and at subsequent service delivery points in the postpartum period on newborn outcomes	**76.0**	70.5	64.6	41.7	54.9	**57.9**	**67.0**
21	postpartum	Describing the psychosocial and mental health characteristics among postpartum women*	**70.0**	83.2	68.6	38.4	63.3	**63.4**	**66.7**
25	postpartum	Investigating the efficacy of tranexamic acid for postpartum hemorrhage prevention	**60.0**	65.5	55.9	40.1	65.7	**56.8**	**58.4**
31	newborn	Investigating the bacterial etiologies of neonatal sepsis and the prevalence of antibiotic resistance*	**81.0**	81.7	77.9	64.6	68.8	**73.2**	**77.1**
30	newborn	Identifying the main causes of neonatal mortality and morbidity*	**74.0**	85.8	77.1	75.0	77.1	**78.8**	**76.4**
28	newborn	Identifying factors and characteristics associated with neonatal growth (weight and length)	**73.0**	77.1	62.9	37.5	68.8	**61.6**	**67.3**
29	newborn	Identifying factors associated with immediate and exclusive breastfeeding among women who gave birth at home compared to women who gave birth at a facility	**64.0**	83.8	61.7	33.3	87.5	**66.6**	**65.3**
32	newborn	Investigating the effect of maternal survival on neonatal survival	**63.0**	74.2	55.0	33.3	79.2	**60.4**	**61.7**
33	newborn	Investigating the effects of immediate and exclusive breastfeeding on newborn outcomes	**56.0**	85.0	62.1	37.5	66.7	**62.8**	**59.4**
35	child	Describing the level of effective coverage of ICCM*	**83.0**	93.8	85.4	69.0	89.6	**84.4**	**83.7**
36	child	Describing the level of effective coverage of IMNCI*	**79.0**	93.8	95.8	59.5	89.6	**84.7**	**81.8**

38	child	Identifying factors associated with childhood immunization drop-out (including for infants, children, and adolescents)*	**80.0**	89.6	87.5	54.8	93.8	**81.4**	**80.7**
40	child	Identifying the main factors contributing to morbidity and mortality of children 1 to 59 months of age*	**74.0**	91.7	83.3	73.8	83.3	**83.0**	**78.5**
42	child	Investigating effective strategies to improve girls' and women's empowerment	**83.0**	79.2	75.0	50.0	81.3	**71.4**	**77.2**
43	child	Investigating factors contributing to immediate initiation and continuation of immunization*	**69.0**	89.6	93.8	56.3	85.4	**81.3**	**75.1**
39	child	Identifying the main factors contributing to morbidity and mortality of adolescents (10 to 19 years of age)	**68.0**	89.6	81.3	73.8	64.6	**77.3**	**72.7**
41	child	Identifying the main factors contributing to morbidity and mortality of children 5 to less than 10 years of age	**67.0**	95.8	77.1	52.1	68.8	**73.4**	**70.2**
34	child	Describing current child immunization coverage levels (including for infants, children, and adolescents)*	**62.0**	91.7	89.6	35.7	91.7	**77.2**	**69.6**
44	child	Investigating the effect of early child stimulation on development/cognition in the first 2 years of life*	**74.0**	84.8	75.0	24.6	69.0	**63.4**	**68.7**
37	child	Describing the prevalence of HIV among children (5 to less than 10 years of age)	**54.0**	79.2	81.3	50.0	79.2	**72.4**	**63.2**
48	health system	Investigating the resiliency of MNCH service delivery/utilization in light of shocks (e.g. pandemics)*	**84.0**	93.8	77.1	50.0	77.1	**74.5**	**79.2**
45	health system	Describing current diagnostic (laboratory and clinical) capacities of the MNCH health workforce*	**75.0**	97.9	81.3	56.3	77.1	**78.1**	**76.6**

47	health system	Identifying effective strategies for improving health facility documentation of MNCH outcomes such as gestational age, preterm birth, facility births, hypertension and (pre)eclampsia*	**80.0**	95.8	83.3	40.5	66.7	**71.6**	**75.8**
46	health system	Describing the level of health facility readiness, including equipment/material availability for MNCH services*	**65.0**	91.7	81.3	54.8	68.8	**74.1**	**69.6**
49	urban rural	Describing care-seeking behavior across different geographies (urban and rural)	**68.0**	91.7	91.7	59.5	77.1	**80.0**	**74.0**
53	urban rural	Describing the usage of over-the-counter, private, and traditional medicines*	**74.0**	75.0	75.0	54.8	77.1	**70.5**	**72.2**
51	urban rural	Describing food taboos, including fasting, for pregnant and lactating women across different geographies (urban and rural)*	**71.0**	85.4	70.8	40.5	75.0	**67.9**	**69.5**
52	urban rural	Describing the private health system and urban/rural population differences in seeking care from private health care providers	**71.0**	87.5	72.9	26.2	83.3	**67.5**	**69.2**
56	urban rural	Investigating the relationship between air pollution exposure and MNCH outcomes*	**70.0**	85.4	68.8	35.7	64.6	**63.6**	**66.8**
54	urban rural	Investigating formula feeding practices and their effects on newborn outcomes across urban/rural populations*	**63.0**	81.3	77.1	54.8	64.6	**69.4**	**66.2**
50	urban rural	Describing current consumption levels of processed foods (e.g. snack foods, soft drinks)	**61.0**	91.7	75.0	26.2	47.9	**60.2**	**60.6**
55	urban rural	Investigating how Cesarean section rates compare between urban and rural populations	**51.0**	89.6	79.2	54.8	54.2	**69.4**	**60.2**

*Note: No. indicates the order in which the research question was presented to respondents. * denote research questions prioritized by the expert consensus discussion groups.*

S2. Expert Consensus Discussion Ranked Priorities by MNCH Domain

Research Question
**Women's Health**
1. Describing the prevalence of gestational diabetes
2. Identifying current behaviors, barriers and supports for antenatal care (ANC) adherence
3. Identifying qualitative and quantitative factors associated with facility delivery versus home delivery
4. Investigating the effect of the availability of WASH in health facilities on maternal and newborn outcomes
5. Assessing the contribution of maternal diet and nutrition during pregnancy to maternal and newborn outcomes
**Intrapartum Health**
1. Identifying the main causes of mortality during the intrapartum period
2. Investigating the effectiveness of intrapartum antibiotic prophylaxis in preventing early-onset group B streptococcal disease
3. Investigating health providers' ability to correctly identify neonates who need to be resuscitated
**Postpartum Health**
1. Identifying barriers to health care-seeking during the postpartum period
2. Investigating the effect of family-centered/family-integrated newborn care on postpartum outcomes
3. Describing the psychosocial and mental health characteristics among postpartum women
4. Describing the level of effective coverage of continuum of KMC in facilities and communities
**Newborn Health**
1. Investigating the bacterial etiologies of neonatal sepsis and the prevalence of antibiotic resistance
2. Identifying the main causes of neonatal mortality and morbidity
**Child Health**
1. Identifying the main factors contributing to morbidity and mortality of children 1 to 59 months of age
2. Describing the level of effective coverage of ICCM/IMNCI
3. Describing current child immunization coverage levels (including for infants, children, and adolescents) and factors associated with immediate initiation, continuation, and drop-out.
4. Investigating the effect of early child stimulation on development/ cognition in the first 2 years of life
**Health System**
1. Describing the level of health workforce readiness, including equipment/material availability for MNCH services and current diagnostic (laboratory and clinical) capacities of the MNCH health workforce
2. Identifying effective strategies for improving health facility documentation of MNCH outcomes such as gestational age, preterm birth, facility births, hypertension and (pre)eclampsia
3. Investigating the resiliency of MNCH service delivery/utilization in light of shocks (e.g. pandemics)
4. Assessing the impact of effective implementation of national strategy & guidelines on key MNCH outcomes
**Urban Rural Differences**
1. Describing food taboos, including fasting, for pregnant and lactating women across different geographies (urban and rural)
2. Investigating formula feeding practices and their effects on newborn outcomes across urban/rural populations
3. Describing the usage of over-the-counter, private, and traditional medicines
4. Investigating the relationship between air pollution exposure and MNCH outcomes

S3. Community Prioritization of MNCH Issues by Theme and Focus Group

**Women's & Maternal Health**	
Angolela Group 1	1. Prenatal micronutrient supplementation for the youth
	2. Impact of health facility distance on service utilization
	3. Balanced diet during pregnancy; the knowledge and practice of pregnant mothers
Angolela Group 2	1. Poor GA Estimation for pregnant mothers with no recollection of LMP
	2. What causes such a low coverage of PNC in the population?
	3. What healthy prenatal alimentation can be advised using the local food?
	4. What options can we use to improve better counseling during pregnancy?
Angolela Group 3	1.How can awareness and demand be improved for early ANC attendance?
	2. Not knowing the expected date of delivery for birth preparedness and avoiding delay at home
	3. PPH due to delay at home
	4. Lack of awareness resulting in the practice of unbalanced diet
Angolela Group 4	1. Food insecure families with pregnancy complications delivering small babies
	2. Relationship between spontaneous abortion and an implant, a longterm FP method
	3. Patriarchal and heavily influencing parenting in the area and lack of disclosure from the teens causing school dropout and early pregnancy
Kewot Group 1	1. Delay in initiating early ANC
	2. Incomplete ANC & Immunization visits and follow ups
	3. Poor maternity waiting room service utilization
Kewot Group 2	1. Sparsely populated with terrain and lack of roads causing poor access to health services
	2. Poor ANC follow up
	3. Bleeding during pregnancy and postpartum period (APH & PPH)
Kewot Group 3	1. Early marriage causing maternal health problems
	2. Quality of health services is low in most health facilities.
	3. Low awareness for the benefits of ANC and PNC utilization
Kewot Group 4	1. Unable to use the MWH facility due to social reasons
	2. Lack of clarity and awareness of services provided at health facility
	3. Bad experience of care at health facilities
**Delivery (Intrapartum)**	
Angolela Group 1	1. Lack of awareness on the care mothers receive during delivery and postpartum.
	2. Poor quality and non-compassionate care from health providers
	3. Delayed care seeking
Angolela Group 2	1. Poor GA and EDD estimation
	2. Shortage of delivery kits at health facility
	3. Poor access to health facility due to distance, economic, or road access reasons
Angolela Group 3	1. Bleeding after home delivery
	2. Teenage pregnancy and its complications
	3. Bad experience of care.
Angolela Group 4	1. Forced home delivery and postpartum bleeding
	2.Mothers choosing home delivery over facility delivery due to home chores
	3. Performing traditional rituals after delivery is easier with home birth than facility birth
Kewot Group 1	1. PPH
	2. Eclampsia-Preeclampsia
	3. Congenital Anomalies
Kewot Group 2	1. Intrapartum and postpartum hemorrhage
	2. Poor access to health facility due to distance
	3. Lack of drugs, supplies, and equipment
Kewot Group 3	1. Lack of transportations/ambulance
	2. Mothers preferring home delivery

	3. Poorly skilled health providers
Kewot Group 4	1. Poor access to health facility due to distance and mothers preferring home birth
	2. Poor referral for blood transfusion
	3. Bad experience of care during delivery
**Newborn health**	
Angolela Group 1	1. Poor knowledge about the benefit of PNC and poor care.
	2. Poor resuscitation skills
	3. Poor facility, skilled man power, and supplies for neonatal care
Angolela Group 2	1. Poor neonatal immunization
	2. What causes high neonatal mortality post delivery?
	3. What causes breathing difficulty in almost all neonates?
	4. What is the local factor that makes neonates vulnerable for pneumonia?
Angolela Group 3	1. Failure to initiate suckling breast milk
	2. Poor access to health facility due to lack of transportation means
	3. Asphyxiation of neonates due to home or on the way to facility delivery and poor health professional attention
Angolela Group 4	1. Skipping birth doth vaccines by parents
	2. Community believes “sore throat/pharyngitis” being common during the neonatal period and seeks traditional remedy causing delay in care seeking
	3. Community believes a “newborn sprain” being common and seeks traditional remedy causing delay in care seeking
Kewot Group 1	1. LBW
	2. Applying foreign materials like dung, butter etc to newborn umbilicus
	3. Asphyxia
Kewot Group 2	1. Preterm birth
	2. Home delivery
	3. Delay in initiation of breast feeding
Kewot Group 3	1. Birth asphyxia
	2. Skipping vaccines provided around birth
	3. Harmful neonatal feeding practice; discarding colostrum, prelacteal feeding of water, tea or butter
Kewot Group 4	1. Child malnutrition
	2. Keeping infants away from light and in the dark, poor thermal care
	3. Poor knowledge and practice of early breastfeeding initiation
**Child Health**	
Angolela Group 1	1. High number of children who did not complete vaccination
	2. Poor hygienic practices leading to high number of infectious diseases
	3. Little behavioral changes on balanced diet and nutrition after so many years of SBC work
Angolela Group 2	1. Malnutrition and food insecurity
	2. Delayed care seeking for sick child
	3. Incomplete knowledge from health professional side to explain the effect of introducing food other than breast milk before the age of six months
Angolela Group 3	1. Poor nutrition leading to wasting
	2. Losing weight after six months due to lack of balanced diet
	3. High number of children who did not complete vaccination causing outbreak of measles
Angolela Group 4	1. Poor feeding practice leading to malnutrition
	2. Poor care provided to children the family
	3. Diarrhea is common especially during the first toothing leading to weight loss
Kewot Group 1	1. Unhealthy dietary habits or nutrition
	2. Ineffective breastfeeding techniques (improper positioning, attachment and suckling)
	3. High prevalence of pneumonia and diarrheal diseases
Kewot Group 2	1. Illegal child trafficking
	2. Harmful traditional practices

	3. Poor utilization of immunization services
Kewot Group 3	1. Lack of awareness to seek immediate health care for those children with danger signs
	2. Mothers not taking their children for vaccination
	3. Poor feeding practice on balanced diet
Kewot Group 4	1. Poor early childhood development (ECD) care exposing to injuries like burn
	2. Poor knowledge and practice of balanced diet mainly due to financial limitations
	3. Poor practice of exclusive breast feeding
	4. Poor hygiene and sanitation leading to skin disease and coughing
**Women in reproductive age**	
Angolela Group 1	1. Poor early ANC initiation
	2. Poor awareness of available services during delivery
	3. Poor awareness of available ANC services
Angolela Group 2	1. Limited preconception care
	2. Poor knowledge on nutrition and food
	3. Early marriage, fast divorce, and its impacts
Angolela Group 3	1. Abortion following rape
	2. Rape, especially when school is too far
	3. Poor family support for rape victims
Angolela Group 4	1. Early school withdrawal from school to work as a domestic servants in Arab countries
	2. Poor menstrual hygiene management leading to missing school days
	3. Irregular menstruation following the use of family planning drugs
Kewot Group 1	1. Unsafe abortion and termination of pregnancy
	2. Migrating illegally to Arab countries
	3. Risky sexual behaviors
Kewot Group 2	1. Rape
	2. Poor knowledge on reproductive health knowledge
	3. Poor preventive practices of HIV and other STI
Kewot Group 3	1. Early marriage and its health complications
	2. Poor awareness on vaccines and screening for cervical CAf
	3. Unsafe abortion and its complications
Kewot Group 4	1. Unwanted teenage pregnancy causing them to leave the area
	2. Fistula following obstructed labor
	3. Delayed care seeking for complications following early pregnancy
